# Immunization of *Mastomys coucha* with *Brugia malayi* Recombinant Trehalose-6-Phosphate Phosphatase Results in Significant Protection against Homologous Challenge Infection

**DOI:** 10.1371/journal.pone.0072585

**Published:** 2013-08-28

**Authors:** Susheela Kushwaha, Prashant Kumar Singh, Ajay Kumar Rana, Shailja Misra-Bhattacharya

**Affiliations:** Division of Parasitology, CSIR-Central Drug Research Institute, Lucknow, Uttar Pradesh, India; University of Oklahoma Health Sciences Center, United States of America

## Abstract

Development of a vaccine to prevent or reduce parasite development in lymphatic filariasis would be a complementary approach to existing chemotherapeutic tools. Trehalose-6-phosphate phosphatase of *Brugia malayi* (Bm-TPP) represents an attractive vaccine target due to its absence in mammals, prevalence in the major life stages of the parasite and immunoreactivity with human bancroftian antibodies, especially from endemic normal subjects. We have recently reported on the cloning, expression, purification and biochemical characterization of this vital enzyme of *B. malayi*. In the present study, immunoprophylactic evaluation of Bm-TPP was carried out against *B. malayi* larval challenge in a susceptible host *Mastomys coucha* and the protective ability of the recombinant protein was evaluated by observing the adverse effects on microfilarial density and adult worm establishment. Immunization caused 78.4% decrease in microfilaremia and 71.04% reduction in the adult worm establishment along with sterilization of 70.06% of the recovered live females. The recombinant protein elicited a mixed Th1/Th2 type of protective immune response as evidenced by the generation of both pro- and anti-inflammatory cytokines IL-2, IFN-γ, TNF-α, IL-4 and an increased production of antibody isotypes IgG1, IgG2a, IgG2b and IgA. Thus immunization with Bm-TPP conferred considerable protection against *B. malayi* establishment by engendering a long-lasting effective immune response and therefore emerges as a potential vaccine candidate against lymphatic filariasis (LF).

## Introduction

Lymphatic filariasis (LF), a mosquito-borne parasitic disease caused by the three species of tissue dwelling filariids, *Wuchereria bancrofti, Brugia malayi and B. timori* is endemic in 72 countries, particularly in the tropics and sub-tropics [1]. An estimated 130 million people living in endemic areas either exhibit disease manifestations or carry microfilariae in their blood [1]. There is a need to discover alternative methods to combat LF in view of negligible or low macrofilaricidal (adulticidal) activity of the available drugs [2], [3], [4], re-appearance of infection after treatment, development of resistance to ivermectin and albendazole and severe drug toxicity in people with heavy loasis infections [5], [6], [7], [8], [9], [10], [11]. An emerging treatment approach against filariasis is the elimination of *Wolbachia* endosymbiont in filarial nematodes by antibiotic treatment [12], [13]. Thus a likelihood approach to lessen transmission and complement MDA (Mass Drug Administration) programs could be the discovery of an effective vaccine [14], [15]. The feasible concept of developing an antifilarial vaccine originates from the endemic normal individuals who despite of being continuously exposed to infective mosquito bites remain infection-free [16]. In addition, successful immune protection has been attained in animal models after vaccination with irradiated infective larvae (L3) [17], [18]. Successful completion of Phase 1 clinical trials of the first human hookworm vaccine showed favourable results [19]. The movement of a schistosomiasis vaccine *Sm-*TSP-2 in to manufacturing process also emphasizes that discovery of an effective filarial vaccine is a viable option [www.sabin.org/programs/schistosomiasis-vaccine]. In a recent study, immunization with a multivalent, subunit vaccine reduced the onset of full patent infection in natural bovine model of *Onchocerca* demonstrating that prevention of patent infection by immunization may be an achievable goal [20]. In the past, a number of *B. malayi* proteins have been identified as vaccine candidates and some of these offered significant degree of protection against infection in animal models [21], [22], [23], [24], [25], [26], [27], [28], [29].

The present study reports on the immunoprophylactic assessment of purified recombinant *B. malayi* trehalose-6-phosphate phosphatase (TPP) in an experimental animal model, *Mastomys coucha.* Bm-TPP has been cloned, expressed and purified to homogeneity as a soluble ∼60 kDa protein. On biochemical characterization, the recombinant protein showed unusual phosphatase activity [30]. Administration of Bm-TPP in BALB/c mice generated a mixed Th1/Th2 immune response that significantly hampered the survival of infective larvae [31]. Since the murine model (BALB/c) used in earlier study does not support the complete life cycle of *B. malayi*, the vaccine potential of Bm-TPP in the present study has been explored in *Mastomys coucha (M. coucha),* the permissive host for *B. malayi*. *M. coucha* accommodates the full cycle of parasite from L3 to the release of Mf in the host blood facilitating assessment of effect of immunization on parasite growth and development. Immunization with Bm-TPP conferred considerable protection against *B. malayi* establishment by generating a long-lasting effective immune response.

## Materials and Methods

### Expression and Purification of *B. malayi* Recombinant Trehalose-6-phosphate Phosphatase

The protein was expressed as described earlier [30]. In brief, the *tpp* coding sequence [Bm1_08695] was amplified from cDNA of adult worms, cloned into the expression vector pET 28a which was further transformed in *E. coli* (DE3) competent BL21. The recombinant protein was purified through Ni-NTA column and dialyzed to remove salts. LPS contamination in the purified protein was <1 EU/mg as determined by toxin sensor limulus amebocyte lysate (LAL) assay kit (Sigma, UK).

### Animals, Immunization and Challenge Infection

Purpose-bred, parasite naive, six week old, male *M. coucha* were used in the study that were maintained in proper housing condition at the Laboratory Animal Division of CSIR-Central Drug Research Institute (CDRI), Lucknow, India and fed on standard pellet diet and water *ad libitum*. The Animal Ethics Committee of CDRI duly constituted under the provisions of CPCSEA (Committee for the Purpose of Control and Supervision on Experiments on Animals), Government of India, approved the animals and the animal experimental procedures. The study bears the Institutional Animal Ethics Committee (IAEC) no. 86/09/Para/IAEC dated 27/4/09. Animals were divided into three groups; Bm-TPP immunized group, adjuvant control group and phosphate buffered saline (PBS) group. Animals were immunized as described earlier [31]. In brief, Bm-TPP immunized group of animals received the first dose (25 µg) of protein emulsified in Freund’s complete adjuvant (FCA; Sigma, USA) by the subcutaneous route followed by two booster doses of the same amount of protein in FIA on days 15 and 21 post 1^st^ immunization. The adjuvant control group received equivalent amount of FCA/FIA in PBS while PBS control group received PBS only. A week following the final booster, *M. coucha* were challenged subcutaneously with 100 *B. malayi* L3. The parameters were acquired in three separate experiments with 4 or 5 animals per group and the data were acquired in blind fashion.

### Assessment of Microfilaraemia, Worm Recovery and Female Worm Fecundity

Mf monitoring was initiated in *M. coucha* beginning on day 90 post L3 challenge (p.c.) and continued monthly up to day 180 by preparing thick blood smears as reported earlier [32]. Briefly, 10 µl tail blood smears were prepared and stained with 2% Giemsa and Mf counted microscopically. On day 180 p.c., the animals were euthanized and adult worms were isolated from various tissues and counted. Arithmetic means were calculated for the total worm burden and the percentage protection was calculated as [(u−v)/u×100], where ‘u’ is the mean value for the control group and ‘v’ is the mean value for the experimental group. Each female worm was teased on glass slide in a drop of PBS and the intrauterine contents of females such as eggs, embryo and Mf were observed microscopically to assess the effect of immunization on worm fecundity. Females having distorted eggs (showing increased space between egg shell and embryo), degenerated embryo, dead or distorted Mf or no Mf in their uteri were considered sterile. Percentage sterilization was calculated as ‘the number of sterile female worms/total female worms teased×100.

### Bm-TPP Specific Serum IgG and Antibody Isotypes by ELISA

Blood for serum was collected from the retro-orbital plexus of *M. coucha* just before L3 challenge and at monthly intervals thereafter. Serum IgG antibody was measured on days 0, 15 and 30 since initiation of immunization and on days 30, 60, 90, 120, 150 and 180 post challenge by indirect ELISA as described earlier [32]. In brief, the ELISA plates (Nunc, Denmark) were coated with Bm-TPP (1 µg/ml), blocked and reacted with Bm-TPP immunized *M. coucha* serum as the primary antibody (1:400) and rabbit anti mouse-IgG-HRP (1:10000) as the secondary antibody (Sigma, USA). Absorbance was read at 492 nm by ELISA reader (Tecan, Switzerland) after adding the substrate orthophenyldiamine (OPD, Sigma) and stopping reaction with 2.5 M H_2_SO_4._

Antibody isotypes were determined by antibody isotyping kit following the manufacturer’s protocol (Sigma, USA) as described earlier [32]. Briefly, plates were coated with Bm-TPP (1 µg/ml), blocked, reacted with the serum samples collected on days 30, 60 and 180 post challenge (p.c.). Goat anti-mouse IgG1, IgG2a, IgG2b and IgA (1:1000) were used as secondary antibodies. Bound isotype specific antibodies were detected after adding HRP conjugated rabbit anti-goat IgG and further processing was done as above.

### Preparation of Anti Bm-TPP Antibody Depleted Immunized *M. coucha* Serum

Bm-TPP specific antibodies from immunized serum were depleted after repeated bindings with Bm-TPP-coupled Ni-NTA agarose resin as described earlier [33]. Briefly, 1 mg of his-tagged Bm-TPP was coupled to Ni-NTA resin overnight in a tube at 4°C. Resin was washed to remove unbound TPP and incubated with 200 µl of immunized serum at 4°C for overnight. After incubation, the resin was washed, centrifuged and the supernatant was collected. The antibody titre was determined by ELISA as described above (Fig. S1). This antibody binding was repeated two times till the supernatant revealed no reaction with Bm-TPP in ELISA. Anti-Bm-TPP antibodies depleted serum was then used in antibody inhibition and ADCC assay.

### Effect of Anti-Bm-TPP Antibodies on TPP Enzyme Activity

Immunized *M. coucha* serum was used to ascertain the inhibition of enzymatic activity of Bm-TPP by anti-Bm-TPP antibody. Bm-TPP was incubated separately with 10 and 20 µl of antibody depleted, antibody intact or normal *M. coucha* serum for 20 min at 37°C in 96 well plate. The reactions were performed in 50 µl volumes containing 50 mM Tris (pH 7.0), 5 mM MgCl_2_, 10% glycerol, 0.5 mM dithiothreitol, 2 mM substrate trehalose-6- phosphate and the recombinant enzyme (0.1 µg) at 37°C for 10 min. Two volumes of filtered solution containing 0.15% malachite green (w/v), 1% ammonium molybdate (w/v) and 12.5% concentrated HCl (v/v) were added and the incubation was continued for another 2 min for colour development. O.D. was taken at 640 nm and the readings were compared amongst various groups.

### Effect of Anti Bm-TPP Antibody on in vitro Cellular Adhesion and Cytotoxicity (ADCC)

Cellular adhesion to Mf and L3 was carried out as described earlier [22]. 100 live Mf and 10 L3 each were co-cultured with 1×10^6^ PECs isolated from normal *M. coucha* in 96 well plates in the presence of normal, immunized and antibody depleted immunized *M. coucha* serum. Plates were kept at 37°C in a CO_2_ incubator and examined microscopically for cellular adherence and cytotoxicity to parasites. Parasite viability was determined under a microscope after 48 h of incubation. If more than 20 cells were attached to the surface of microfilariae and >5 cells to infective larvae then these were considered as showing ‘cell adherence’. The larvae that were limpid, damaged and immobile were considered to be dead. The percentage cytotoxicity was estimated using the formula.

Number of dead larvae ÷ Total number of larvae × 100.

### Isolation of Splenocytes

On day 180 p.c. animals were euthanized and the spleens were surgically removed. Single cell suspensions of splenocytes were prepared by passing through nylon filter (70 µm) and rendered free of RBCs after lysis with chilled Tris-ammonium chloride, washed, counted and suspended at desired concentration.

### Extracellular Cytokine Estimation

Splenocytes suspensions (100 µl/well) from stock (5×10^6^ cells/ml) were plated in 96 well Nunc culture plates in triplicate, stimulated with 100 µl/well of Con A (2.5 µg/ml) and 100 µl of Bm-TPP (2.5 µg/ml). The supernatant was collected after 48 h and cytokines (Th1: IFN-γ, TNF-α, IL-2 and Th2: IL-4, IL-10) in the cell culture supernatant were quantified using mouse Th1/Th2 cytometric bead array (CBA) kit (BD Biosciences Pharmingen, San Diego, CA) following manufacturer’s protocol.

### Immunofluorescence Assay (IFA) to Detect Bm-TPP on Mf and L3 Stage

The reactivity of murine polyclonal antibodies raised against Bm-TPP with Mf and L3 was investigated by immunofluorescence microscopy. MF were recovered from the peritoneal cavity of infected gerbils, made free of host tissue, passed through 5.0 µm membrane filter, thoroughly washed and pelleted after and repeated washing. L3 were recovered from the infected mosquitoes as mentioned earlier and washed several times in sterile PBS. Ten L3 and 50 Mf were incubated with serum at a final concentration of 10% in RPMI for 1 h at 37°C in 48 well flat-bottom tissue culture plates. The parasites were washed and re-incubated in secondary antibody (antimouse IgG-FITC) for 2 h at room temperature on a shaker. After washing, the parasites were transferred to glass slide for fluorescence microscopy (Nikon, Japan).

### In Vitro Lymphoproliferation

Single cell suspensions of splenocytes were prepared and plated (100 µl/well) in the same way as for cytokine estimation. Cells in triplicate were stimulated with 100 µl (2.5 µg/ml) each of Bm-TPP or LPS or Concanavaline A for 72 h and pulsed with 1.0 µCi/well of [^3^H] thymidine (^3^H-Tdr, specific activity 18Ci/m mole, BARC, India) for 18 h preceding harvest. The radioactive incorporation was measured in a standard liquid scintillation counter (Beckman Instruments, CA) and stimulation indices (SI) were calculated as mean cpm (counts per minute) of stimulated culture/mean cpm of unstimulated culture.

### Statistical Analysis

Data were analyzed using one or two way analysis of variance (ANOVA) with the help of statistical software PRISM 5. Individual comparisons following ANOVA were made using the Newman-Keuls method. The criterion for statistical significance between the groups was as follows: *p* value <0.05 was considered significant and marked as *, <0.01 as highly significant and marked as **, <0.001 as very highly significant and marked as ***.

## Results

### Bm-TPP Specific Antibody Inhibits Bm-TPP Enzyme Activity

**Figure 1:**
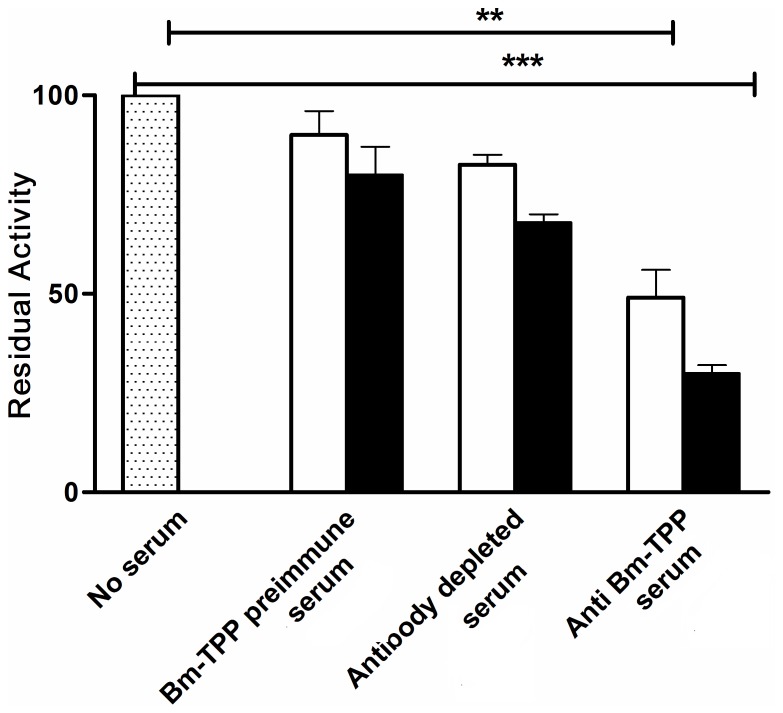
Reduction in Bm-TPP phosphatase activity. Recombinant Bm-TPP was pre-incubated with 10 and 20 µl (represented as open bar and closed bar respectively) of anti Bm-TPP sera and with pre-immune sera. Significant reduction in activity was noticed with anti Bm-TPP sera. Bar represents the mean ± S.E. of three replicate experiments. Statistical significance based on the differences between the mean values of immunized and control sera are indicated as *p<0.05; **p<0.01 and ***p<0.001.

Anti-Bm-TPP serum significantly inhibited the enzyme activity by 51% and 70% respectively at 10 and 20 µl per reaction (*p*<0.01–0.001). Depletion of Bm-TPP specific antibody significantly (∼17–32%) reduced the inhibitory activity of anti-Bm-TPP sera. Incubation with pre- immunized or control sera did not show any significant (*p*>0.05) inhibition (8%) in the enzyme activity (Fig. 1).

### Anti Bm-TPP Antibody Induces Adherence of PECs and Cytotoxicity to L3 and Mf

**Figure 2:**
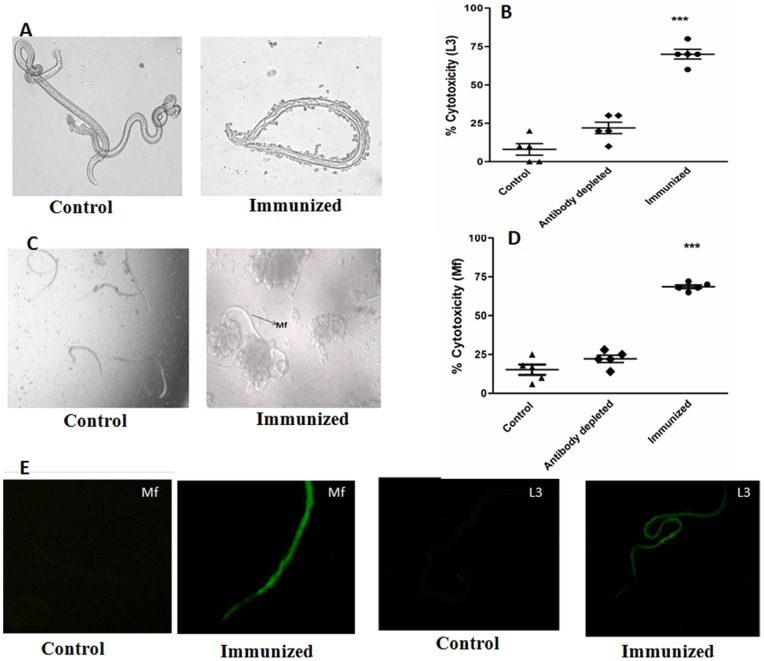
Antibody dependent cellular adhesion to L3 and Mf of *B. malayi*. Mf and L3 were incubated with peritoneal exudates cells in the presence of anti Bm-TPP sera. Significant cellular adhesion on the surface of (**A**) L3 and (**C**) Mf was observed which caused death of parasite with in 48 h as a result cytotoxicity (**B**) L3 and (**D**) Mf. Photographs were captured on a phase contrast microscope (Nikon, Japan). Data are presented as mean ± S.E. of six replicate of experiment. Statistical significance based on the differences between the mean values among the groups are indicated as *p<0.05; **p<0.01 and ***p<0.001. (**E**) Interaction of anti Bm-TPP antibodies with *B. malayi* infective larvae (L3), and microfilariae (Mf) is demonstrated by indirect fluorescence. Parasites were incubated with anti Bm-TPP sera and further incubated with FITC labelled anti-mouse IgG. Images were captured under a fluorescent microscope at 20X for Mf, 10X for L3. Serum from naive animals at similar dilution was used as control and no detectable fluorescence could be seen in any of the above parasite stages.

Serum of Bm-TPP immunized animals promoted significant adherence of PECs to the surface of L3 and Mf causing 70±3.7% and 65±1.2% cytotoxicity and death respectively within 48 h as opposed to those incubated with serum from non immunized animals (8±3.2% and 15.2±3.3% respectively) (Fig. 2A–D). Removal of anti-Bm-TPP antibodies from the immune serum reduced (*p*>0.05) this cytotoxic effect to 22%±3.1 and 21%±2.3 respectively (Table S1). IFA results showed that antibodies bind to the surface of both Mf and L3. The intensity of binding was found higher in Mf. No detectable binding at the same dilution of pooled serum of naïve animal was noticed, however (Fig. 2E).

### Bm-TPP Triggers Profound Antibody Secretion in Sera of *M. coucha*

**Figure 3:**
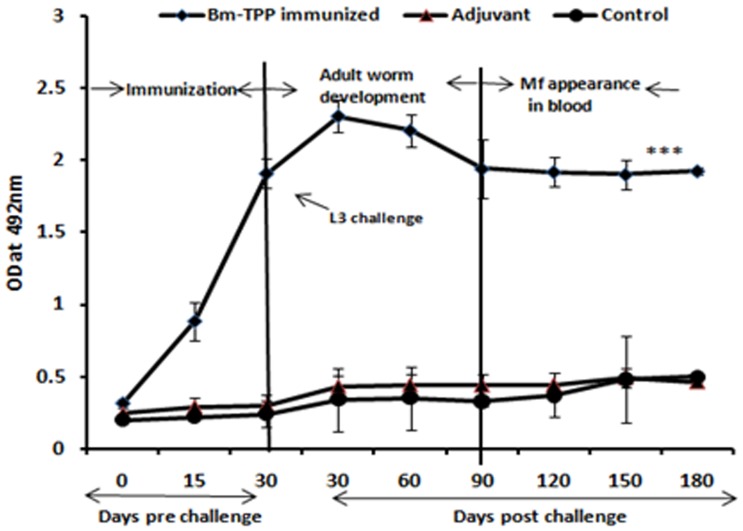
Anti-Bm-TPP antibody level measurement. Anti-Bm-TPP antibody levels were measured in the sera of control and immunized *M. coucha* before and after L3 challenge by ELISA. High Bm-TPP specific IgG titer was generated in immunized animals that remained high throughout the observation period while control and adjuvant groups of animals did not generate anti-Bm-TPP antibodies.

**Figure 4:**
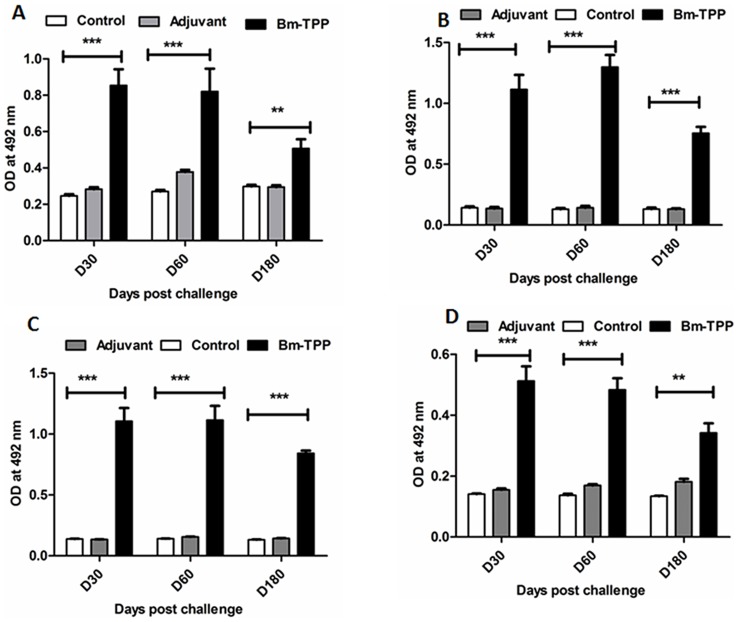
IgG isotypes and IgA antibody levels in *M. coucha*. IgG isotypes and IgA antibody levels were measured at 30, 60, 180 day post-challenge in all the three groups. (**A**) High levels of IgG1, (**B**) IgG2a, (**C**) IgG2b and (**D**) IgA antibodies were found at all time points**.** Bars represent the mean ± S.E (n = 13). The statistical significance based on the differences between the mean values of immunized and control groups are indicated as *p<0.05; **p<0.01 and ***p<0.001.

Bm-TPP immunized animals produced high titres of specific IgG antibody as compared to control groups (*p*<0.001) which remained elevated till day 180 p.c. (Fig. 3). Immunized animals had significantly raised levels of IgG2a (*p*<0.001) and IgG2b (*p*<0.001) followed by IgG1 (*p*<0.001) isotype (Fig. 4A–C) and IgA (*p*<0.01) (Fig. 4D). The antibody titres declined slightly with the onset of microfilaremia but still remained significantly higher over those of controls (*p*<0.01 to<0.001).

### Bm-TPP Vaccination Causes Significant Reduction in *B. malayi* L3 Establishment, Adult Worm Recovery and Female Worm Fecundity

**Figure 5:**
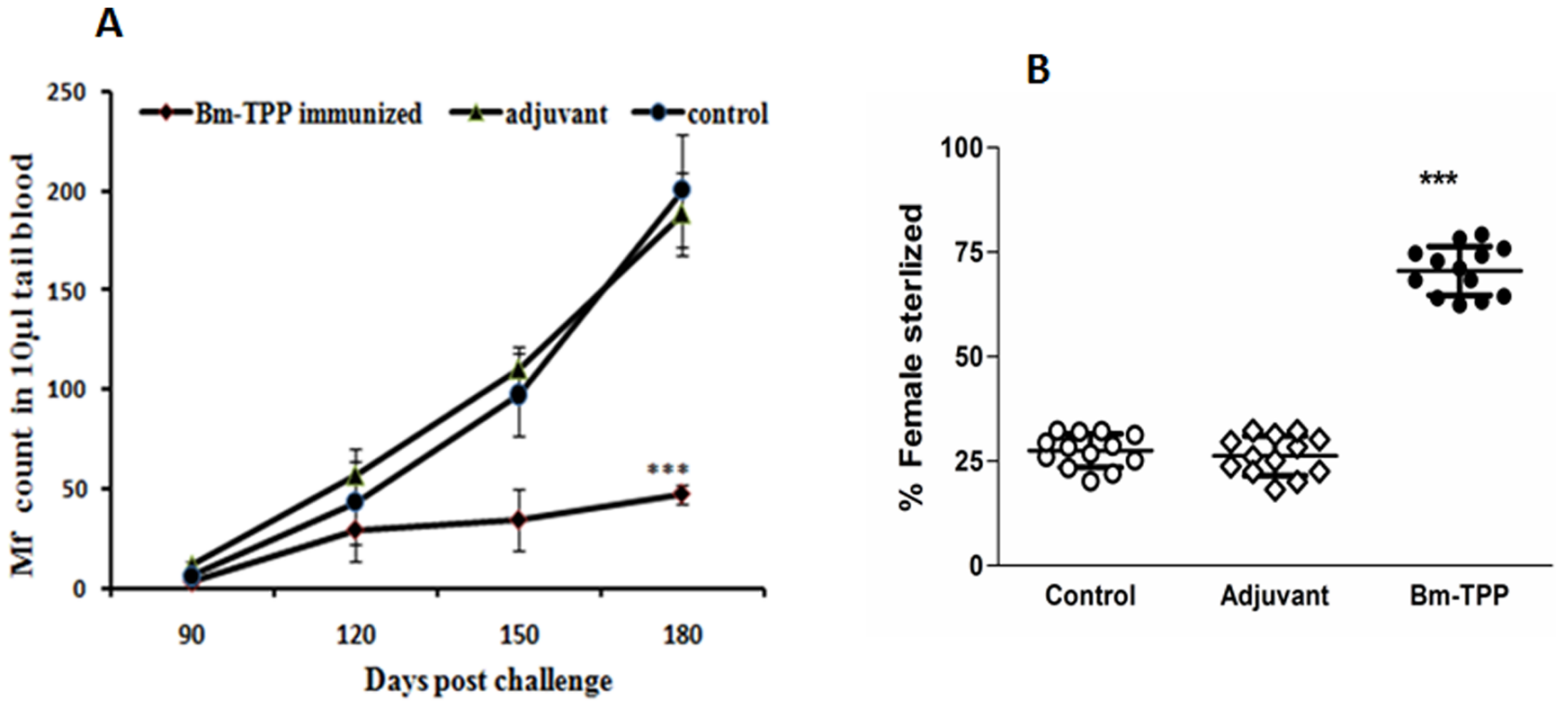
Immunization resulted in to significant reduction in parasite burden. (**A**) Microfilaraemia in the tail blood of immunized and control groups of *M. Coucha* were determined after 90 days post challenge. Significant reduction in microfilarial density was observed in Bm-TPP immunized *M. coucha* throughout the observation when compared to PBS and adjuvant controls. (**B**) Sterility of female worms was determined by observing the intrauterine contents of female on day 180 post challenge. The female bearing degenerated eggs, embryo, mf were considered sterile. Vaccination with Bm-TPP induces significant sterilization of female worms. Statistically significant percentage of females recovered from immunized mastomys was found sterilized. One dot represents the percentage of female worms recovered from one animal. Bars represent the mean ± S.E. (n = 13) and the statistical significance based on the differences between the mean values of immunized and control groups are indicated as *p<0.05; **p<0.01 and ***p<0.001.

A 78% reduction of microfilaremia (*p*<0.001) occurred in the vaccinated group on day 180 p.c. (Fig. 5A). The recovery of both male and female adult worms was found to be much lower in Bm-TPP immunized animals (71.04% reduction; *p*<0.001) (Table 1). In addition, significant number of female worms recovered from vaccinated *M. coucha* were sterile i.e. these contained degenerated eggs, embryo and stretched Mf (70.06±2.5%; *p*<0.001) *in utero* (Fig. 5B) (Fig. S2).

**Table 1:** Adult worm recovery from different groups.

Animal groups	No. ofanimals	Adult worm count/Animal	Adult worm recovery (mean±S.E.)	% reduction in worm burden
**PBS immunized**	13	♀ = 30,24,22,17,21,15,23,22,23,20,15, 19,21; ♂ = 9, 8,7,11,8,9,7, 7,7,6, 12, 5,5	28.69±3.9	–
**Adjuvant immunized**	13	♀ = 22,21,18,22,12,23,21,22, 20,24,15,18,17; ♂ = 13,8,11,8,7,9,6,9, 6,4,11,8,9	27.23±3.2	5.08
**Bm-TPP immunized**	13	♀ = 7,8,7,5,6,4,6,8,6,7,5,4,3; ♂ = 4,2,2,3,2,5,3,1,3,2,1,2,2	8.30±1.7	71.04***

*** *p*<0.001 value significant difference from control group, data represent the values from three independent experiments with 4 to 5 animals each group.

### Bm-TPP Elicits a Predominant Th1 Cytokine Response

**Figure 6:**
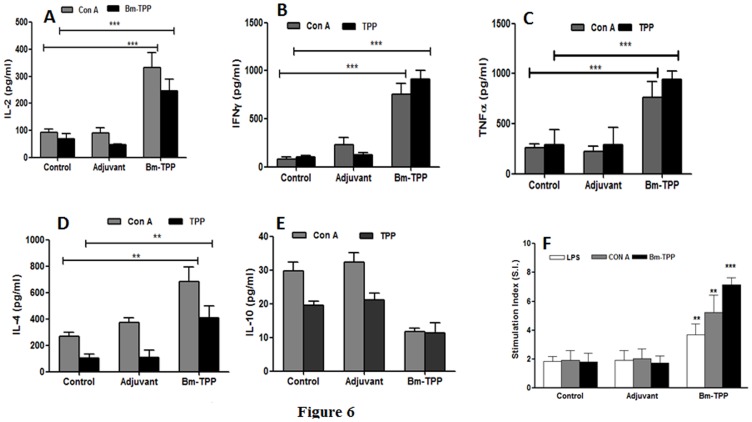
Immunization with Bm-TPP induced heightened immune response in *M. coucha*. Splenocytes harvested from control, infected control and immunized-infected *M. coucha* were stimulated *in vitro* with mitogen Con A (2.5 µg/ml) and Bm-TPP (2.5 ug/ml). Levels of IL-2 (**A**), IFN-γ (**B**), TNF-α (**C**), IL-4 (**D**) and IL-10 (**E**) in the cell free supernatants were measured using cytometric beads by flow cytometry. (**F**) Splenocytes harvested on day 180 post challenge from the control and immunized *M. coucha* were *in vitro* stimulated with Con A (2.5 µg/ml) and Bm-TPP (2.5 µg/ml). Significant cellular proliferation could be observed in the immunized animals even after establishment of patent infection. Bars represent the mean ± S.E. (n = 13). Statistical significance based on the differences between the mean values of immunized and control groups are indicated as *p<0.05; **p<0.01 and ***p<0.001.

Bm-TPP immunized animals demonstrated significantly enhanced release of antigen specific and Con A induced anti- and pro- inflammatory cytokines IFN-γ, IL-2 and TNF-α and IL-4 (*p*<0.05 to <0.001) (Fig. 6A–D). Higher IL-4 content (*p*<0.001) was found to be stimulated by Con A in all the three groups with highest antigen specific release of IL-4 in immunized animals (Fig. 6D) (*p*<0.05). Control animals generated enhanced levels of IL-10 (*p*<0.001), However no significant up-regulation in IL-10 was noticed in the immunized animals (Fig. 6E).

### Bm-TPP Vaccinated *M. coucha* Produced Significant Cellular Immune Response

The *in vitro* proliferative response of splenocytes harvested from recombinant Bm-TPP vaccinated animals on day 180 p.c. revealed much higher lymphoproliferation in presence of Con A and Bm-TPP (*p*<0.01–*p*<0.001) in contrast to cellular hypo-responsiveness demonstrated by two unimmunized L3 challenged control groups in the presence of recombinant protein or mitogen (Fig. 6F).

## Discussion

The mechanism by which filarial parasites promote their survival in immune-competent hosts depends upon the imbalance in Th1 and Th2 cytokines leading to a dominating Th2 immune response. However, there are several other mechanisms involved which include the production of regulatory cytokines, altered function of antigen-presenting cells (APCs) [34], [35], [36], T-cell apoptosis [37] and up-regulation in inducible nitric oxide (NO) synthase (iNOS) [38]. This suggests that a prospective vaccine molecule must be able to evoke both cellular and humoral immune responses. Genes expressed by the parasite at key stages of their development may provide novel targets for developing new immunoprophylactic or transmission blocking agents. We earlier identified and biochemically characterized *B. malayi* trehalose-6-phosphate phosphatase that was found to be expressed in L3, Mf and adult parasites [30]. The recombinant protein also cross-reacted with the human bancroftian sera belonging to various clinical categories (asymptomatic microfilaraemic, symptomatic and endemic normals) [39], [40]. The sero-reactivity of r-Bm-TPP was found specific as non-endemic normal sera from filaria-free zone failed to show any reaction with the recombinant protein [31]. r-Bm-TPP on administration in to BALB/c mouse elicited a mixed Th1/Th2 immune response besides impairing the survival of infective larvae in the peritoneal cavity [31]. In the present study, immunoprophylactic studies have been designed to assess the effect of immunization on parasite development in rodent host that supports the full life cycle of *B. malayi* similar to human infections.

Trehalose has been shown to have copious roles in nematode biology such as in glucose uptake, egg hatching, embryonic development, protecting nematode from various stress conditions [41], [42], [43], [44]. Anti Bm-TPP antibodies significantly inhibited the enzyme activity *in vitro*. *In vivo* inhibition of enzyme activity by specific antibody is likely to impede these functions exposing parasite to stress conditions in the host thereby affecting their establishment and development. In earlier study, we have demonstrated that inhibition of expression of TPP in infective larvae via siRNA significantly impaired the L3 establishment in the host [45] thus demonstrating an important role of Bm-TPP in L3 survival. Anti Bm-TPP antibodies also promoted significant death of L3 *in vitro* during ADCC assay. Depletion of anti Bm-TPP antibodies from the resistant serum, however, significantly reduced the cytotoxicity and larval killing suggesting the possible involvement of anti-Bm-TPP antibodies in the observed protection. ADCC is one of the well known immunological mechanisms operating against filarial parasites both *in vitro* and *in vivo* involving neutrophils, macrophages and eosinophils [46], [47], [48]. In several past studies, it has been shown that the antigen whose antibodies induced cytotoxicity to parasite either *in vitro* or *in vivo* was found to have vaccine potential and brought about significant protection against the filarial infection [25], [33]. The interaction of anti-Bm-TPP antibodies with the parasite was also confirmed by immunofluorescence where anti Bm-TPP antibodies exhibited binding with the surface of parasite throughout the length demonstrating surface presence of Bm-TPP. The immunized *M. coucha* generated a significant IgG antibody response against Bm-TPP in the serum. Several earlier studies have shown a major role of antibodies in protection against LF, and immunity has been transferred passively by transfer of resistant serum thereby strongly suggesting a major role of antibodies in protective immunity [49], [50]. An inverse correlation between adult worm population and protective circulating antibodies to the surface of L3 and Mf has also been accounted [46], [47], [50]. Immunized animals also had low microfilaremia in the blood. One of the reasons for this low microfilaraemia could be the removal of Mf produced by females via ADCC mechanism as noticed in the *in vitro* assay. Another reason might be the low worm burden that resulted in to reduced microfilaraemia in immunized mice. Apart from reduction in parasite burden, large proportion of female worms recovered from the immunized animals were sterile and had either fewer healthy eggs, or mostly degenerated eggs and Mf in their uteri leading to reduced blood microfilaraemia.

Immunization provoked up-regulation in the levels of Bm-TPP specific IgG1, IgG2a and IgG2b antibodies that remained elevated throughout the observation period including patency. Amongst these, IgG2a and IgG2b were more prominent followed by IgG1, reflecting generation of a mixed Th1/Th2 immune response. IgG1 and IgG2a antibodies are reported to offer adequate protection against challenged filarial larvae post irradiated L3 vaccination. Significant increase in Bm-TPP specific IgA antibody isotype was also noticed post L3 challenge after administering the recombinant protein. The protective role of IgA in intestinal helminths has been well worked out [51], [52], [53], [54]. A recent publication indicates negative correlation between the IgA antibody and human filarial infection further affirming its role in providing protective immunity [55].

Significant up-regulation in both Th1 and Th2 cytokine production was observed in immunized animals both in presence of Bm-TPP and Con A. IFN-γ has been found to be essential in host defence for the encapsulation of adult *Litomosoides sigmodontis* in the inflammatory nodules as also for normal worm clearance [56], IL-4 prevents filarial development in resistant mice and aids in the clearance of MF from the blood stream. Moreover, mice deficient in IL-4 exhibit diminished Th2 responses against *Trichuris muris* infection and fail to produce parasite-specific IgG1 [57], [58]. Immunity in human infections has been reported to be associated with an elevated level of IL-2 and IFN-γ [59], [60]. Moreover, IFN-γ is also known to activate macrophages to secrete ROS and NO that are crucial in parasite killing [61]. The results obtained from cytokine analysis which is a better indicator of Th1 or Th2 response, were in accordance with the isotype pattern mentioned above. Control mice infected with *B. malayi* developed a typical Th2 response as revealed by an up-regulated IL-4 and IL-10 associated with impaired secretion of Th1 cytokines such as IL-2 and IFN-γ even in the presence of nonspecific mitogen Con A. This effect was also earlier described in primary infections of both humans and mice (permissive or non permissive) with *Brugia* L3 that resulted in to induction of a Th2 host response [58], [62], [63], [64] and impaired Th1 immune response i.e. complete absence of any antigen specific IFN-γ secretion *in vitro.*

The splenocytes of immunized animals mounted significant cellular proliferation both in the presence of nonspecific mitogen Con A and Bm-TPP. However, the control animals showed hypo-responsiveness even in the presence of Con A. Thus situation of immunized animals is similar to parasite exposed but uninfected individuals who mount higher Th1 response and cellular proliferation in presence of non-parasite or parasite specific antigen. In the present study, immunization with Bm-TPP protected *M. coucha* against the *B. malayi* challenge over a long period of six months. The long-term immunological memory was established as demonstrated by antigen specific antibody level and cellular proliferation till the end of experiment. The presence of a mixed Th1 and Th2 type of immune responses could be one of the reasons for bringing about the facilitated killing of L3 leading to reduced worm recovery from immunized *M. coucha*. FCA/FIA being one of the most powerful experimental adjuvant known so far was used in the current study, however, further investigations are underway with other human compatible adjuvants to produce similar Th1 and Th2 response since both the T helper cell responses appear necessary for eliciting an effective protective immune response against the filarial parasites. Efforts are also in progress to achieve complete protection by the use of multiple immunogenic recombinant proteins in combination.

## Conclusion

The current study deals with the immunoprophylactic evaluation of an immunogenic recombinant protein, trehalose-6-phosphate phosphatase of human lymphatic filarial parasite, *B. malayi.* An attempt was also made to investigate the possible mechanism of immune mediated protection. Vaccination followed by homologous L3 challenge resulted in to a considerable reduction in the establishment of L3 in *M. coucha* with resultant low Mf density, reduced adult worm recovery and profound sterilization of established live female worms. Intense cellular adherence and cytotoxicity to L3 and Mf was mediated *in vitro* by the resistant serum. The protective efficacy correlated perfectly with the augmented humoral and cellular immune responses developed against Bm-TPP.

## Supporting Information

Figure S1 **Bm-TPP antibody depletion from immunized serum.** The serum was incubated with resin coupled with Bm-TPP at 4°C overnight. After incubation, the serum was removed and antibody titer was measured. The serum of first elution was then incubated with fresh Bm-TPP coupled resin in the same manner. This was done for four times after that OD in ELISA became equal to pre- immune serum. For ELISA, The wells of microtiter plate was coated with 100 ng of recombinant protein and serum from different steps were added at 1:100 dilution. The reaction was developed with goat anti-mouse HRP labelled secondary antibody. The pre-immune sera was used as control. X axis label denotes the serum after different incubation steps.
(DOC)

Figure S2 **Intrauterine content of females recovered from control and Bm-TPP immunized animals.** Females from control groups were fertile where their uterus contained various embryonic stages (Figure A) while the uteri of females recovered from immunized group (Figure D) had fewer stages. The eggs of females recovered from control groups had normal phenotype (Figure B) while the eggs from immunized group were degenerated (Figure E). Intrauterine content of control females had eggs, different embryonic stages and Mf (Figure C) while the females from immunized group contained only degenerated eggs or embryonic stages (Figure F).
(DOC)

Table S1 **Percentage of L3 and Mf with attached cells and cytotoxicity.**
(DOC)
