# Supplementary material for: Immunization of Mastomys coucha with Brugia malayi Recombinant Trehalose-6-Phosphate Phosphatase Results in Significant Protection against Homologous Challenge Infection
Source: PLoS One. 2013 Aug 28;8(8):e72585. doi: 10.1371/journal.pone.0072585 (PMC3755969; doi:10.1371/journal.pone.0072585)
Supplement: Figure S1 — Bm-TPP antibody depletion from immunized serum. The serum was incubated with resin coupled with Bm-TPP at 4°C overnight. After incubation, the serum was removed and antibody titer was measured. The serum of first elution was then incubated with fresh Bm-TPP coupled resin in the same manner. This was done for four times after that OD in ELISA became equal to pre- immune serum. For ELISA, The wells of microtiter plate was coated with 100 ng of recombinant protein and serum from different steps were added at 1:100 dilution. The reaction was developed with goat anti-mouse HRP labelled secondary antibody. The pre-immune sera was used as control. X axis label denotes the serum after different incubation steps. (DOC) [file pone.0072585.s001.doc]

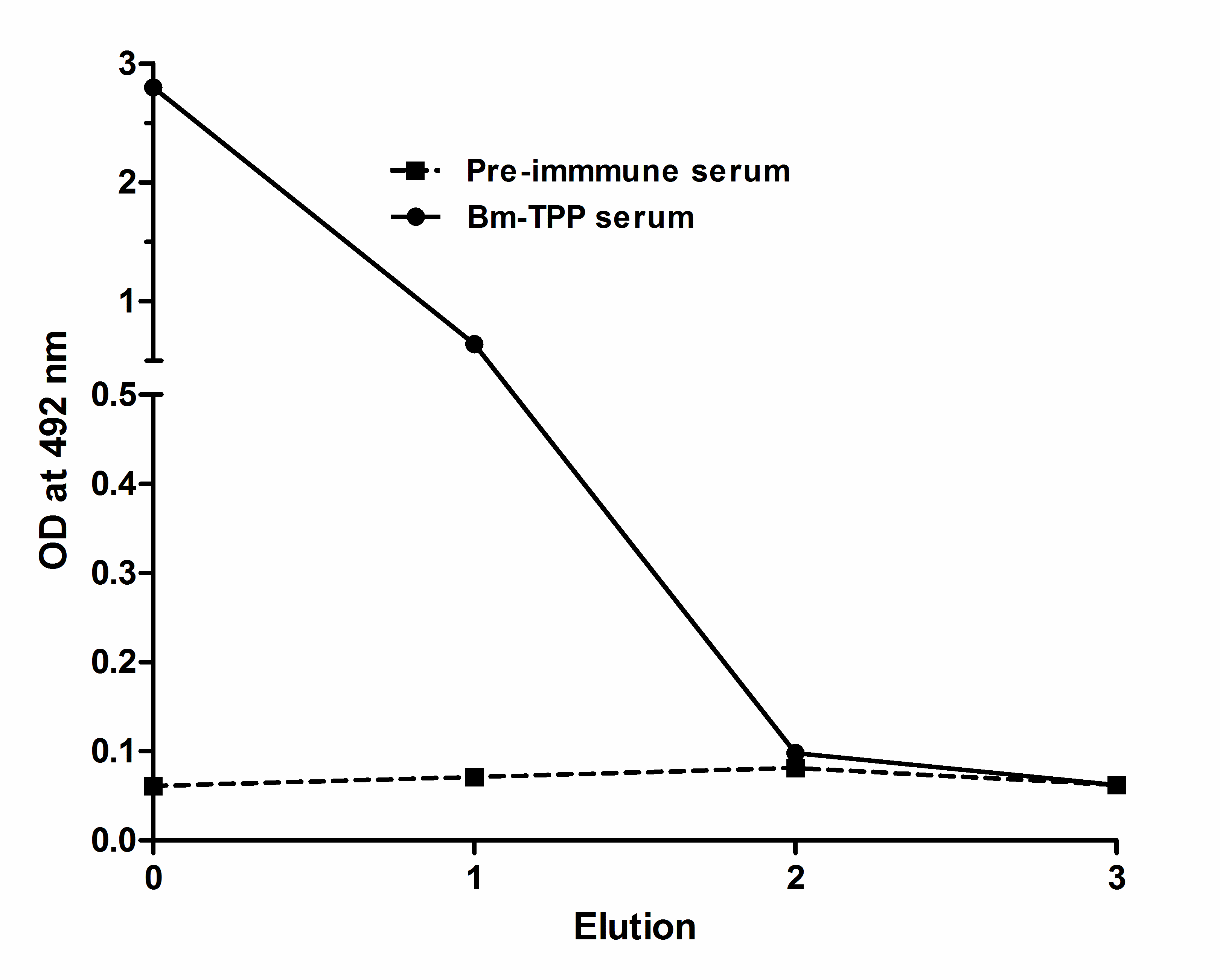


Figure S1: Bm-TPP antibody depletion from immunized serum. The serum was incubated with resin coupled with Bm-TPP at 4^0^C overnight. After incubation, the serum was removed and antibody titer was measured. The serum of first elution was then incubated with fresh Bm-TPP coupled resin in the same manner. This was done for four times after that OD in ELISA became equal to pre- immune serum. For ELISA, The wells of microtiter plate was coated with 100 ng of recombinant protein and serum from different steps were added at 1:100 dilution. The reaction was developed with goat anti-mouse HRP labelled secondary antibody. The pre-immune sera was used as control. X axis label denotes the serum after different incubation steps.
