# Supplementary material for: Immunization of Mastomys coucha with Brugia malayi Recombinant Trehalose-6-Phosphate Phosphatase Results in Significant Protection against Homologous Challenge Infection
Source: PLoS One. 2013 Aug 28;8(8):e72585. doi: 10.1371/journal.pone.0072585 (PMC3755969; doi:10.1371/journal.pone.0072585)
Supplement: Figure S2 — Intrauterine content of females recovered from control and Bm-TPP immunized animals. Females from control groups were fertile where their uterus contained various embryonic stages (Figure A) while the uteri of females recovered from immunized group (Figure D) had fewer stages. The eggs of females recovered from control groups had normal phenotype (Figure B) while the eggs from immunized group were degenerated (Figure E). Intrauterine content of control females had eggs, different embryonic stages and Mf (Figure C) while the females from immunized group contained only degenerated eggs or embryonic stages (Figure F). (DOC) [file pone.0072585.s002.doc]

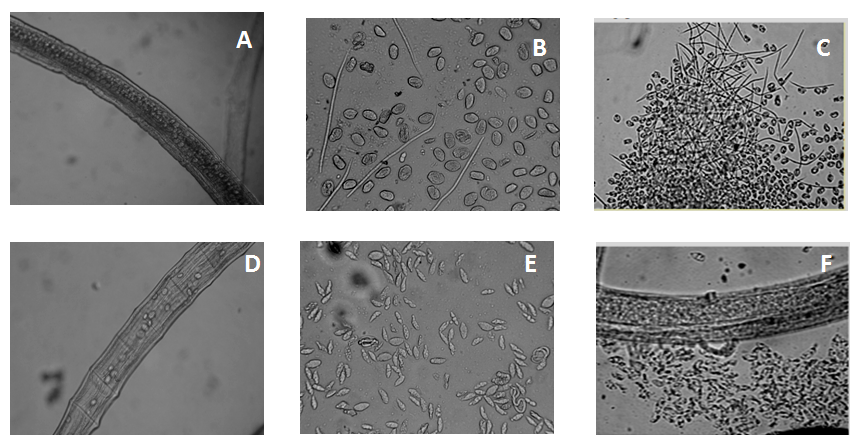


**Figure S2:** Intrauterine content of females recovered from control and Bm-TPP immunized animals. Females from control groups were fertile where their uterus contained various embryonic stages (Figure A) while the uteri of females recovered from immunized group (Figure D) had fewer stages . The eggs of females recovered from control groups had normal phenotype (Figure B) while the eggs from immunized group were degenerated (Figure E). Intrauterine content of control females had eggs, different embryonic stages and Mf (Figure C) while the females from immunized group contained only degenerated eggs or embryonic stages (Figure F).
