# Supplementary material for: Immunization of Mastomys coucha with Brugia malayi Recombinant Trehalose-6-Phosphate Phosphatase Results in Significant Protection against Homologous Challenge Infection
Source: PLoS One. 2013 Aug 28;8(8):e72585. doi: 10.1371/journal.pone.0072585 (PMC3755969; doi:10.1371/journal.pone.0072585)
Supplement: Table S1 — Percentage of L3 and Mf with attached cells and cytotoxicity. (DOC) [file pone.0072585.s003.doc]

Table S1: Percentage of L3 and Mf with attached cells and cytotoxicity.

| **Experimental groups** | **% of L3/Mf with attached cells** | | **% Cytotoxicity** | |
| --- | --- | --- | --- | --- |
|  | L3 | Mf | L3 | Mf |
| **Control** | 12 | 22 | 8 | 15 |
| **Immunized group** | 75 | 90 | 70 | 65 |
| **Antibody depleted** | 25 | 28 | 21 | 22 |
